# Isolation, Purification and Antioxidant Activity of The Polysaccharides from Chinese Truffle *Tuber sinense*

**DOI:** 10.22037/ijpr.2020.1100954

**Published:** 2020

**Authors:** Caihong Zeng, Xiaoyan Chen, Wenwen Jiang, Yanling Liu, Chunjuan Fang

**Affiliations:** a *Jiangxi University of Technology, Nanchang 330098, Jiangxi, China. *; b *Center of Drug Metabolism and Pharmacokinetics, China Pharmaceutical University, Jiangsu 210009, China.*

**Keywords:** Purification, Molecular structure, Tuber sinense, Antioxidant activity, Total reducing capacity

## Abstract

The content of polysaccharides in *Tuber sinense* was investigated by isolation and purification, followed with the further antioxidant studies in total reducing capacity and radical scavenging activities. The crude extract of polysaccharides was purified by dialysis, column chromatography, and High Performance Liquid Chromatography. The main components of monosaccharide (s) and molecular structure of single polysaccharide were studied by using methylation, GC-MS, and NMR analysis. One new water-soluble non-starch polysaccharide (PTS-A with the yield of 0.41%) from *T. sinense* was purified and identified on structural characteristics for the first time. The characterizations of PTS-A were studied on physicochemical properties, main components of monosaccharide (s) and molecular structure. PTS-A was identified as glucan, only containing D-glucoses with the molecular structure of [→6) α-D-Glcp (1→6) α-D-Glcp (1→]_n_ by methylation analysis and NMR. In the determination of total reducing capacity, their reducing abilities could be listed as vitamin C> PTS-A> crude polysaccharides-3> crude polysaccharides-2> crude polysaccharides-1. All of PTS-A, crude polysaccharides-2 and -3 were relatively good scavenger for 1,1-Diphenyl-2-picrylhydrazyl radical 2,2-Diphenyl-1- (2,4,6-trinitrophenyl) hydrazyl radicals with the IC_50_ of 2.81, 4.17 and 3.44 mg/mL, respectively. Thus, the separation and purification of polysaccharides were significant to increase the antioxidant activity in some degree. One new water-soluble 1,6-α-ᴅ-dextran was discovered with the polysaccharide structure identified for the first time. Both PTS-A and crude extracts of polysaccharide performed a potent potential on antioxidant activities. The bioactivities of PTS-A should be generalized to the broader pharmacological effects.

## Introduction

As a medicinal and edible fungus parasitizing on the trees, the wild Chinese truffle *Tuber sinense* (Tuberaceae, Ascomycota) is popular in folk as food for many years, due to its highly nutritional attribute. *T. sinense*, the class of black truffle or *Perigord truffle*, is also obtained as experimental subject these years. Numerous polysaccharides and protein-polysaccharide complexes extracted from *T. sinense* were utilized widely as a source of therapeutic agents for the treatment of tumors, anti-inflammatory, and immune activities (1-4). The polysaccharide of *T. sinense* (PST) is a protein-bound polysaccharide first extracted from the Chinese truffle, and it has been utilized widely for the treatment of tumors. It was reported that PST dramatically inhibit the growth of S180 sarcoma and Ehrlich’s ascites carcinoma sarcoma *in-vivo*, but there was no related effect on cell proliferation *in-vitro*, which meant that the anti-tumor effect of PST might be related to immunomodulation but not cytotoxic activity. In another side, PST therapy increased the weight of mouse spleen and the level of serum antibodies, which proofed the assumption as well. As a fungous polysaccharide with low toxicity and good water solubility, PST showed significant anti-tumor potential and might have much more medicinal value. The research group of Tang YJ *et al*. aimed to not only increase the production of extracellular polysaccharides in the plantation area of medicinal mushroom Chinese truffle *Tuber sinense* (3), but also separated more than fifty-two polysaccharides from the fermentation systems of *T. melanosporum*, Tuber indicum, Tuber sinense, Tuber aestivum, and the fruiting bodies of Tuber indicum, Tuber himalayense, Tuber sinense by elution with an activated carbon column. The polysaccharides from Tuber fermentation system exhibited relatively higher *in-vitro* antitumor activity against HepG2, A549, HCT-116, SK-BR-3, and HL-60 cells than those from Tuber fruiting bodies (4). Besides, the specific aromas of *T. sinense* had been exploited on plenty of special pharmacological effects, such as sexual performance improving, anti-tumor, anti-aging, and so on (5,6,3).

As a medicinal and edible fungus parasitizing on the trees, the wild *T. sinense* was obtained as experimental subject by our research team for several years. Sixty-five main compounds of the aroma profile of *T. melanosporum* were identified, including most components of alcohols and lipids by GS-MS (7). According to the previous study on the new water-soluble non-starch polysaccharide (PTS-A) from *T. sinense*, it was demonstrated that the molecular weight of PTS-A was 7.29×10^5^ Da approximately (8). Their physicochemical properties and compositions were studied to provide reference and basis for the main pharmacological activity of truffle and its corresponding mechanisms. Therefore, the systematic study on its components of aromas and polysaccharides will be meaningful for its potential utilization with broad market prospect.

*Experimental*

*The source of the truffles*

**Figure 1:**
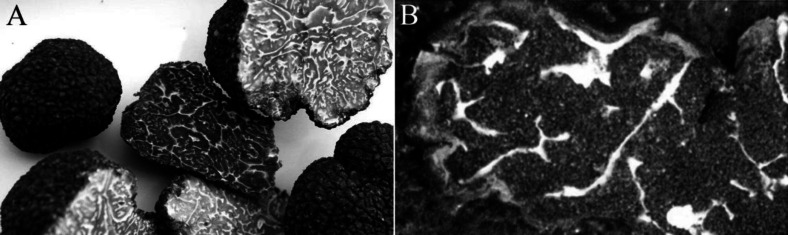
The raw samples of Chinese truffle *T. sinense* (A: cross section; B: the venation of colonies) being used for identification

The dry and raw material of Chinese truffle *T. sinense* (ref no. HMAS60222) is collected in Oct 2015 from Huidong city of Sichuan province in China (9). All sources, identified by Dr. Jun-xuan Yang (Chengdu University of TCM) as shown in Figure 1, were frozen after vacuum-packed. The outer peridial layer was pseudoparenchyma and composed of subglobose to ellipsoid cells 10-30 µm diam. The asci usually contained 1-7 (8) spores ornamented with spines connected by low ridges to form an alveolate reticulum. The reticulum was usually regular and less than 1 µm tall, and the spine was 5-7 µm tall. In most cases, *T. sinense* have spores ornamented with sparsely or densely free spine (5-10 across the spore width), and some cases have spores with incomplete reticulum or ridge formed by spine weakly connected each other at the base, which can differs it from *T. indicum* on the ascospores surfaces formed by spines.

*Polysaccharide purification and purity identification*

To prepare the powder sources, the dry and raw materials were grinded and sieved through 100 meshes. According to our optimal condition of sonication extraction, the ultrasonic power is 105 W, followed with the solid-liquid ratio of 25: 1 in distilled water for 40 min at 75 °C. After the 4500 rpm centrifugation for 15 min, the crude polysaccharides-1 was obtained as supernatant. The crude polysaccharides-2 was also collected after deproteinization by Sevag method (10). Next, the crude PTS was concentrated and performed with dialysis (30 × 1000 mm) in water for 72 h, and in distilled water for next 12 h. The crude polysaccharides-3 was collected and sequentially purified by chromatography of DEAE-52 and Sephadex G-100 according to the reported methods with some modifications (11). Briefly, the crude polysaccharides-3 (5 mL, 20 mg/mL) was loaded on a Cellulose DEAE-52 column (2.6 cm × 40 cm) equilibrated with deionized water. PTS was then fractionated and eluted with distilled water and different concentrations of stepwise NaCl solutions (0.2, 0.4 and 0.6 M) at a flow rate of 1 mL/min. Two fractions were collected by checking the absorbance at 490 nm by using the phenol-sulfuric acid method (12). The major one of the two fractions were further fractionated by size-exclusion chromatography on a Sephadex G-100 column (1.6 × 50 cm) and eluted with deionized water at a flow rate of 0.5 mL/min to afford PTS-A. Finally, the purified PTS-A were collected, concentrated, and lyophilized for further study. The yields of samples in each part were calculated after freeze drying. The purity of PTS-A was detected by Shimadzu HPLC with RID-10AT detector (differential detection). Pump was IC-10AT with a Cosmosil amino column (4.6 × 250 mm). The injection volume was 20 μL at 25°C with flow rate of 0.5 mL/min, and the wavelength of detection was at 197 nm with the mobile phase of a mixture of methanol-ultra-pure water (20: 80).

*Structural characteristics of polysacch-arides*

*The compositions of monosaccharide*

At first, 20.0 mg of polysaccharides were dissolved in 2 mL of 2 mol/L trifluoroacetic acid. The reaction solution was sealed, and fully hydrolyzed at 100 °C for 8 h. The reaction vessel was removed out and cooled down to room temperature. After centrifugation at 1000 rpm for 5 min, the supernatant was neutralized with NaOH to pH 7.0 and freeze-dried as the hydrolyzates of polysaccharides.

The monosaccharide derivatives were prepared before HPLC analysis. 80 μL of each standard monosaccharide solution (0.2mol/L) was accurately measured and mixed in one tube, followed by adding 100 μL of 0.3 mol/L NaOH and 1.2 mL of 0.5 mol/L 1-phenyl-3-methyl-5-pyrazolone, dissolved in methanol. The mixture was denatured at 70 °C water bath for 40 min, then cooled down to room temperature and neutralized with HCl. Thereafter, it was extracted for three times with 1 mL CHCl_3_, the upper aqueous phase contained the derived products of monosaccharide. The derivatives for hydrolyzates of polysaccharide (PTS-A) were also done according to the method mentioned above (13, 14).

HPLC-UV detection was conducted in Agilent 1100 chromatographic system with Waters Symmetry C18 column (150×4.60 mm) at the detecting wavelength of 245 nm. The injection volume was 20 μL with flow rate of 1.0 mL/min at 40 °C. The mobile phase was a mixture of ammonium acetate buffer (CH_3_COONH_4_-CH_3_COOH, modified to pH 6.0 with acetic acid) and acetonitrile in a ratio of 80: 20 (v/v).

*Infrared spectroscopy of polysaccharide*

One milligram of each sample was mixed with certain amount of potassium bromide (grinded and dried) in the metal mold. After pressurizing for 5 min, the dispersed transparent tablet could be obtained and put on the stent of infrared spectrometer, scanning in the wavelength range of 500-40000 cm^-1^. The results were recorded.

*PTS-A methylation analysis*

Two milligram of PTS-A was accurately weighed and vacuum dried at 70 °C for 3 h. 2 mL of anhydrous dimethyl sulfoxide was added and stirred till dissolved. 1.8 mol/L of methyl sulfinyl anion was added and followed with nitrogen stream. Abundant precipitations were showed, but disappeared and dissolved again after stirring. 1 mL of methyl iodide was added dropwisely into the solution, which was kept in room temperature below 30 °C and stirred until a clear pale yellow showed. After reaction stopped 1 h later, the methylation products were filled in a dialysis bag to dialyze in the flowing water flow for 24 h. Then it was concentrated and freeze-dried for use. Its methylation level could be checked by IR spectrum. Methylation should be repeated if it is not complete.

Subsequently, the methylated polysaccha-ride was hydrolyzed and followed with acetylation. The 1mL formic acid was added into certain amount of methylated polysaccharide, which was in an Abe tube full of nitrogen. It was put in 100 °C oven to hydrolyze for 6 h. After that, formic acid was moved and followed by 0.5 mL of 2 mol/L trifluoroacetic acid to hydrolyze methylated polysaccharide again in the same process. The products were resolved in 0.5 mL water and deoxidized by 2 mg sodium borohydride at 30 °C for 4 h. The next step was acetylation that was realized by pyridine and acetic acid (15). The acetylation products were dissolved in 1mL of methanol. After the organic membrane filtration, the solution can be used for GC-MS analysis.

The GC column was Agilent 122-2932 DB225 (0.25 mm×30 m×0.25 μm) followed with MS detector MSD. Temperature program began with initial temperature of 110 ^o^C. The heating rate was 7 °C/min, and the temperature was kept at 230 °C for 18 min. The temperature at injector was 230 °C. Carrier gas was helium. Injection volume was 1 μ with 1.0 mL/min flow rate of carrier gas. According to the data of spectra in standard CCRC database, the connecting bond-type of sugar would be confirmed.

*NMR analysis of PTS-A*

PTS-A of 15 mg was dissolved in 0.5 mL D_2_O, using TMS as an internal standard. ^13^C-NMR and ^1^H-NMR spectrums were measured by the Bruker AM-400 MHz superconducting NMR instrument.

*Anti-oxidative activity in-vitro*

*Determination of total reducing capacity*

It was conducted by ferric reducing ability assay (16). Three mililiters of phosphate buffer (pH 6.8, 0.2 mol/L) and 2.5 mL of 1% potassium ferricyanide (K_3_Fe (CN)_6_) solution were added into the polysaccharide solutions with different concentration. After rapid mixing 45 °C water bath for 30 min, immediate cooling and adding 3.0 mL of 10% trichloroacetic acid (TCA) solution, the solution was centrifuged at 6000 rpm for 20 min. Three mililiters of the supernatant was mixed with 2.0 mL of distilled water and 1.0 mL of 0.1% ferric chloride (FeC1_3_) solution, and measured for its absorbance at 700 nm wavelength. The higher absorbance means the reducing power is stronger. In the comparison with distilled water as negative control and vitamin C (VC) solution as positive control, the experiment was repeated three times for each sample and the average value was calculated.

*Determination of 1,1-Diphenyl-2-picrylhydrazyl radcal 2,2-Diphenyl-1- (2,4,6-trinitrophenyl) hydrazyl (DPPH) radical scavenging activity*

According to the reference (17), the reaction system included 1.0 mL of polysaccharide solution with different concentrations, Two mililiters of 0.2 mmol/L DPPH-ethanol solution and Two mililiters of 95% ethanol. All were mixed well, reacted in the dark for 30 min. The absorbance was measured at a wavelength of 517 nm. 2.0 mL of 95% ethanol solution was used instead of DPPH as a blank sample. The solution in control group was 2.0 mL of DPPH solution mixed with 3.0 mL of 95% ethanol. Vitamin E (VE) was applied as positive control. The lower absorbance of the reaction system, indicates the stronger DPPH radical scavenging activity. The rate of DPPH radical scavenging is calculated according to the formula as follows. I/%= (A_o_- A_1_)*100)/ A_o_. I/% represents the percentage of the clearance rate; A_1_ is the absorbance of the sample group; A_0_ means the absorbance of the control group. All samples were tested in triplicate, and the average value was calculated.

*Determination of O*_2_^—^ *• clearing ability*

Using the pyrogallol autoxidation method (18), pyrogallol in alkaline conditions can cause autoxidation. To each tube containing 6.0 mL of Tris-HCl buffer (50 mmol/L, pH = 8.1), Half a mililiter of polysaccharide solution with different concentrations was added. The mixture is in the water bath of 37 °C for 10 min. Then l.0 mL of the hydrochloric acid solution of pyrogallol (7 mmol/L) was added. The mixture is in the water bath of 37 °C for 10min. Then l.0 mL of the hydrochloric acid solution of pyrogallol (7 mmol/L) was added, shaken, and reacted precisely for 4 min. After that, the reaction was quenched with 0.5 mL of concentrated HCl. The absorbance was measured at a wavelength of 325 nm. The distilled water was used as blank sample, and VC as positive control. The O_2_^—^• clearing ability was estimated based on the formula following. I/%= (A_o_- A)*100)/ A_o_. I/% represents the percentage of clearance rate; A_o_ is the absorbance of control group; A indicates the absorbance of sample solution. All the samples were tested in triplicate, and the average value was calculated.

*Statistical Analysis*

The *t* test was used to make comparisons between the mean values of independent samples. The analysis was performed by applying the SPSS statistics system (version 16.5). Significance was defined as *p* < 0.05 and the variables are presented as the mean ± SD.

## Results

*The yield of polysaccharides (dry weight)*

The yields of crude polysaccharides-1, -2, -3, measured by phenol-sulfuric acid method, were 3.48%, 2.13% and 1.79%, respectively. The yield of pure PTS-A was 0.41%. In this process, three crude polysaccharides were obtained and attempted to compare their anti-oxidization abilities with PTS-A.

**Figure 2:**
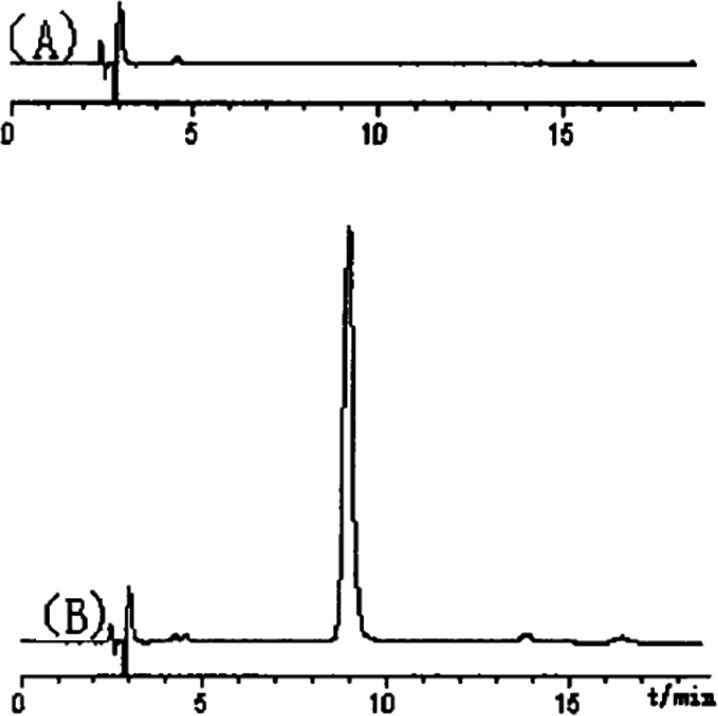
Purity identification of truffle polysaccharide (PTS-A) by HPLC-UV (A: blank, B: PTS-A).

The carbohydrate polymer (PTS-A) might be isolated as neutral polysaccharides. After the purification of SephadexG-100 column chromatography, the main peaks were collected, concentrated, and finally analyzed by HPLC-UV (Figure 2). In our previous study on molecular weight of polysaccharides [8], several dextran standards with different molecular weights got different retention time (RT) in the same HPLC conditions. So, according to the relationship between RT and molecular weight, the formula can be obtained as Ig (M) = -0.0817RT+ 6.587. The molecular weight of PTS-A was calculated as 7.29×10^5^ Da separately.

*Structural characteristics of polysaccharides*

*Molecular weights and monosaccharide compositions of PTS-A*

**Figure 3:**
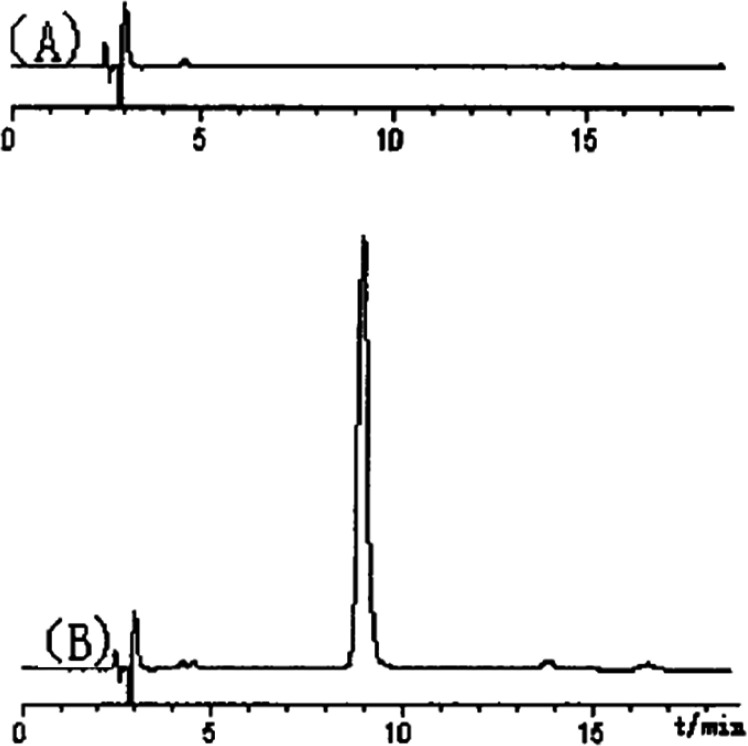
HPLC spectrum of acetylated aldononitrile derivatives of (a) mixed standard monosaccharide; (b) PTS-A

PTS-A gave a single peak, indicating that it was homogeneous polysaccharide. By the quantitative method with external standard, PTS-A was glucan only containing D-glucoses (Figure 3), which indicated that the PTS-A were homopolysaccharide. Its average molecular weight was estimated to be 728 kDa.

*The methylation analysis of PTS-A*

The sample of PTS-A was fully methylated, followed by complete acid hydrolysis, and finally made into acetylated derivatives for GC-MS analysis, and the results were shown in Table 1. Referencing the literature from Guo. (15), the only derivative of 1,5,6-tri-O-acetyl-2,3,4-tri-O-methyl-D-glucitol could be detected obviously in PTS-A. It could be inferred that PTS-A was mainly composed of (1→6)-linked glucose group.

*The FT-IR spectra and NMR analysis of PTS-A*

The FT-IR spectra of PTS-A displayed a broad stretching intense characteristic peak at 3400 cm^−1^ for the hydroxyl group and a weak C-H band at around 2930 cm^−1^. The relatively strong absorption peaks at around 1638 cm^−1^ (C=O asymmetric stretching vibrations), 1400 cm^−1^ and 1385 cm^−1^ (C=O symmetric stretching vibrations) indicated that the PTS-A was acidic polysaccharides (19). The peaks toward 1000-1200 cm^−1^ suggested the presence of C-O-C and C-O-H link bonds, indicating the presence of pyranose. A characteristic band approximately at 816 cm^−1^ was due to α-ᴅ-Glc (20).

**Figure 4:**
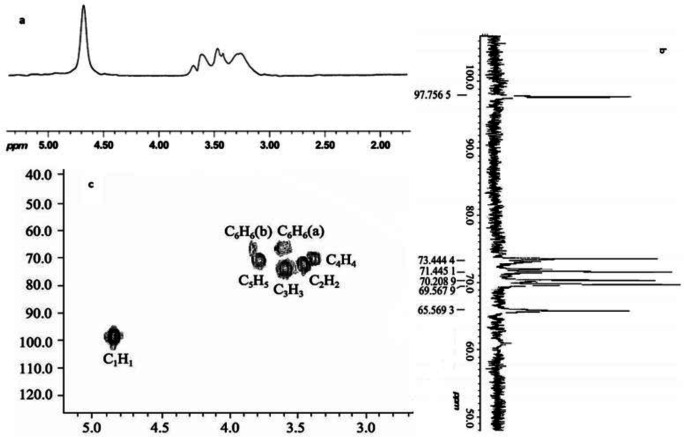
The (a) ^1^H-NMR, (b) ^13^C-NMR and (c) HMQC spectra (D_2_O, 500 MHz) of PTS-A

As shown in Figure 4, NMR analysis was carried out to further verify the molecular structure of PTS-A (15). There were six peaks with basically equal height in PTS-A ^13^C-NMR spectrum. From low-field to high-field they were 97.74, 73.42, 71.43, 70.21, 69.57, and 65.59. The chemical shift of 97.74 was the signal peak of the C1 on the terminus (21). It was approved again that PTS-A was composed of α-D-glucose and identical with the results of the infrared spectra. There was signal peak of C6 substitution at 65.59, with no other signal peaks at 80.00. So it could be concluded that there was no C2, C3, or C4 substitution existing. This result was consistent with the methylation (22). The experimental results of ^1^H-NMR and ^13^C-NMR showed that PTS-A might be a linear glucan connected by α-D-(1→6), and its structure could be determined as follows: [→6) α-D-Glcp (1→6) α-D-Glcp (1→]_n_.

*In-vitro antioxidant activity*

*Determination of total reducing capacity*

**Figure 5:**
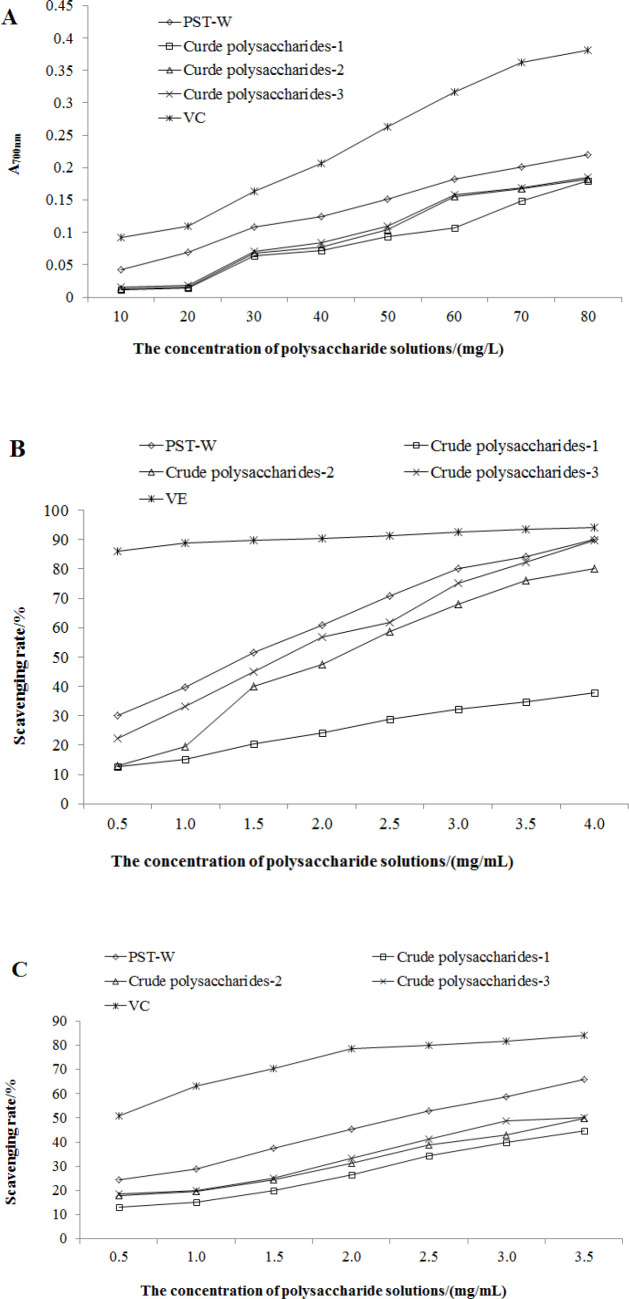
Antioxidant activities of PTS-A and crude polysaccharides (A: Total reducing power; B: DPPH radical scavenging activity; C: Superoxide anion radical scavenging activity)

Considering that the reducing ability is obviously positive related to the antioxidant activity, the absorbance of the reactive products at 700 nm indicates the intensity of reducing ability. The results of each sample were showed in Figure 5A. It seemed that, along with the purification procedure of crude polysaccharide, the crude extract with higher purity inferred to a stronger reducing power. So their antioxidant effects could be listed as VC> PTS-A> crude polysaccharides-3> crude polysaccharides-2> crude polysaccharides-1. The activities of three crude polysaccharides were similar and weak.

*Determination of DPPH radicals scaveng-ing activity*

DPPH radicals are a kind of stable aromatic radicals. The scavenging activity of antioxidants on DPPH is generally acknowledged as the total ability on clearing radicals (23). All samples showed their abilities on scavenging DPPH radicals (Figure 5B). When the concentration of polysaccharides samples was 4.0 mg/mL, the clearance rates of PTS-A, crude polysaccharides -2, and -3 were 90.2%, 80.1%, and 89.8%, close to the 94.1% of VE. The clearance ability of crude polysaccharides-1 at 4.0 mg/mL also achieved 37.9%.

*Determination of O*_2_^—^ *·* *clearing ability*

O_2_^—^· is the first one of all oxygen radicals, and can produce other oxygen radicals through series of reactions. It is highly toxic, and the clearing capacity for extra O_2_^—^· is seemed as particular significance (24). Compared with VC, the clearing ability of all crude polysaccharides samples were relatively weak (Figure 5C). It is surmised that polysaccharide might only react with O_2_^—^· by its hydrogen on reactive hydroxyl, which could not inhibit the product of O_2_^—^· fundamentally.

*Value of IC*_50_

Each assay on antioxidant activity was reproductive and repeatable. According to Table 2, the antioxidant capacities of PTS-A and crude polysaccharides on scavenging DPPH and superoxide anion radical were calculated in the form of IC_50_. It indicated that all of PTS-A, crude polysaccharides-2, and -3 were relatively good scavenger for DPPH radicals with the IC_50_ of 2.81, 4.17, and 3.44 mg/mL, respectively. However, the O_2_^—^· clearing abilities of PTS-A and crude polysaccharides were obviously weaker than their capacities on DPPH.

**Table 1:** The GC-MS results of methylated PTS-A

**Methylated sugar residue**	**Retention time (min)**	**Major ion peak of MS (m/z)**		**Glycosidic bond chaining**
		**PTS-A**	**References (15)**	
2,3,4 –Me_3_-Glc	25.239	101,117,129,161,189,233	101,117,129,161,189,233	→6) Glc (1→

**Table 2:** IC_50_ values of PTS-A and crude polysaccharides for scavenging DPPH and superoxide anion radical (mg/mL).

**Samples**	**DPPH radical**	**O**_2_^—^ **·**
PTS-A	2.81±0.008	4.73±0.017
Crude polysaccharides-1	6.48±0.041	7.55±0.036
Crude polysaccharides-2	4.17±0.020	7.01±0.027
Crude polysaccharides-3	3.44±0.012	6.56±0.015
VE	0.15±0.003	—
VC	—	0.87±0.002

Means ± SD (n = 3) followed by ^*^, ^**^, ^***^ are significantly different from values obtained for Crude polysaccharides-1 (^*^p <0.05;

^**^p < 0.01; ^***^p < 0.001; T test).

## Discussion

In recent research (25), five miligram per mililiter polysaccharide extracts of *Perigord Truffle* (PEPT) demonstrated its high scavenging abilities on hydroxyl radicals and DPPH with the EC_50_ of 0.73 mg/mL and 1.12 mg/mL, respectively. In the study against redox reaction induced by potassium ferricyanide, ferric chloride and trichloroacetic acid, the EC_50_ of PEPT was 2.46 mg/mL. In another study, PEPT was divided into three groups according to molecular size by dialysis membrane: TIP-III) group included the ingredients of PEPT with molecular weight >100 Da; TIP-II group contained components of PEPT with molecular weight between 50~100 Da; TIP-I group was compositions of PEPT with molecular weight <50 Da (26). The TIP group with smaller molecular weight resulted in higher clearance and stronger antioxidant activities on DPPH radicals, •OH, O_2_^—^•and iron ions (TIP-III>TIP-II>TIP-I).

To the best of our knowledge, this was the first investigation on the chemical characteristics, monosaccharide composition, and antioxidant activities of polysaccharide PTS-A from Chinese truffle *T. sinense*. PTS-A is the main soluble polysaccharide of truffle. In this study, our results indicated the presumed molecular structure of PTS-A was [→6) α-D-Glcp (1→6) α-D-Glcp (1→]_n_, wherein the value of n could be calculated to be about 2×10^3^. In the determination of total reducing capacity, their reducing abilities could be listed as VC> PTS-A> crude polysaccharides-3> crude polysaccharides-2> crude polysaccharides-1. All of PTS-A, crude polysaccharides-2, and -3 were relatively good scavenger for DPPH radicals. However, the O_2_^—^· clearing abilities of PTS-A and crude polysaccharides were obviously weaker. For the antioxidant activities of PTS-A and polysaccharide extracts, the activities of total crude extract were the worst, indicating that the impurities might negatively affect the antioxidant activity. Thus, the separation and purification of polysaccharides were significant to increase the antioxidant activity in some degree.

It was proposed that the possible antioxidant mechanism of PTS-A may involve hydrogen donation to break chain reactions, and free radical scavenging ability resulting from the abstraction of anomeric hydrogen from the internal monosaccharide units of polysaccharides (27). Most importantly, the biological activities of polysaccharides are tightly associated with molecular weight, uronic acid, monosaccharide composition, degree of substitution and branching, structure, and conformation. In particular, the antioxidant activities of different polysaccharide fractions were recently correlated positively with the increasing sulfate group content (28, 29) and the decreasing molecular weight of polysaccharides (30, 31). Considering there is no sulfate group in the branch of PTS-A, it may answer for its relative week antioxidant activity.

## Conclusion

It was also demonstrated that Chinese truffle *T. sinense* is a healthcare food and a source of natural antioxidants and further investigation of its antioxidant properties *in-vivo* and other studies of the biological activities of these polysaccharides are in progress. Further studies on their application in the food, medical, and cosmetic industries are worth exploration.
